# Predictive Models of Atherogenic Risk in Citizens of Trujillo (Peru) Based on Associated Factors

**DOI:** 10.3390/nu16234138

**Published:** 2024-11-29

**Authors:** Jackeline del Pilar Bustamante Gallo, Cinthya Stephany Neglia Cermeño, Jorge Luis Díaz-Ortega, Irma Luz Yupari-Azabache

**Affiliations:** 1 Escuela Profesional de Nutrición, Universidad César Vallejo, Trujillo 13001, Peru; jbustamanteg@ucvvirtual.edu.pe (J.d.P.B.G.); cneglia@ucv.edu.pe (C.S.N.C.); 2 Institutos y Centros de Investigación, Universidad César Vallejo, Trujillo 13001, Peru; iyupari@ucv.edu.pe

**Keywords:** cholesterol, triglycerides, lipoproteins, cardiovascular risk, sleep, physical activity, obesity

## Abstract

Background/objectives: Atherogenic risk is related to lipid metabolism imbalance and the likelihood of cardiovascular disease (CVD). The purpose of this study was to determine predictive models based on physiological parameters, family history, and lifestyle for atherogenic risk, assessed by indicators such as total cholesterol (TC)/HDL, triglycerides/HDL, LDL/HDL, and non-HDL cholesterol in citizens of the city of Trujillo (Peru). Methods: A total of 267 people, recruited from September to December 2023, participated in the study. Their lipid profile, glycaemia, abdominal perimeter, and blood pressure were determined, and questionnaires were applied with questions on diet, physical activity, alcohol consumption, smoking, hours of sleep, and family history. Binary logistic regression was considered to determine prediction models for each atherogenic risk indicator. Results: High values were found for all atherogenic indicators; dietary habits were poor in 86.1%; physical activity was low in 35.2%; hours of sleep were less than 7 h in 64.4%; and alcohol and tobacco consumption were low in 8.2% and 9%, respectively. The family history of CVD corresponded to the mother, father, grandmother, and grandfather in 53.2%, 44.9%, 30.3% and 25.1%, respectively. In addition to the inclusion of BMI in the predictive models of atherogenic risk, for the case of total cholesterol/HDL, the variable grandparental history and female sex were included; for TG/HDL, low physical activity, male sex, and alcohol consumption were associated; and for LDL/HDL and non-HDL cholesterol, female sex and age were associated. Conclusion: The best prediction model for atherogenic risk is the corresponding model for TG/HDL, without ignoring the grandfather’s history of CVD and age.

## 1. Introduction

Atherosclerosis manifests as a chronic disease of the arterial wall that culminates in clinical disease through partial or complete obstruction of blood vessels due to lumen narrowing or localized thrombosis. It stands as the leading cause of vascular disease-related mortality worldwide [1]. Atherogenesis is a multifactorial process involving genetic and environmental factors, developing over a long period before showing clinical manifestations (prolonged latency period) and with various risk factors involved; among these factors, lipoproteins and lipids play an essential role [2]. Globally, the prevalence of hypercholesterolemia in adults varies, reaching up to 54% in Europe and 48% in America, while in Southeast Asia and Africa, the prevalences are 30% and 23%, respectively [3]. In Peru, the prevalence of hypercholesterolemia is approximately 20% in the Peruvian population over 20 years old, while hypertriglyceridemia and high LDL levels affect 15% and 13%, respectively [4]. Diet is a modifiable risk factor associated with cardiovascular disease (CVD), and it has been shown that optimal intake of healthy foods, including whole grains, vegetables, fruits, nuts, legumes, dairy products, and fish, reduces the risk of CVD by up to 65%. This risk reduction may be partly due to the anti-inflammatory potential of these foods, as atherosclerosis, the underlying cause of CVD, is a chronic inflammatory disease of the arteries, and healthy food groups have been found to modulate this inflammation [5]. Few hours of sleep (less than 7 h per night) is associated with endocrine changes, modifying food intake with a preference for energy-dense foods. Therefore, fragmented sleep is associated with poor sleep quality, altering lipid and glucose metabolism, as well as cardiovascular regulation [6]. It has been determined that alcohol consumption is associated with increased triglycerides after dinner and at bedtime; however, the timing of alcohol consumption itself is less important than the amount of alcohol consumed, as alcohol promotes an increase in the secretion of very low-density lipoprotein (VLDL) [7]. Regarding tobacco, it has an indirect effect on lipoprotein metabolism, affecting increases in cholesterol and triglycerides, reducing HDL, and decreasing the antiatherogenic effect by altering its composition [8]. The various toxic components of cigarette smoke are responsible for damage to blood vessel walls, causing artery hardening, as they generate oxidative stress by producing oxidized LDL, which, in turn, stimulates an inflammatory response by macrophages in the initiation of atherosclerosis [9].

Central obesity, indirectly related to visceral fat, is strongly associated with atherogenic dyslipidemia. The increase in visceral adipose tissue leads to structural and functional changes in adipocytes characterized by insulin resistance, which, in turn, triggers lipolysis and conversion of triglycerides into free fatty acids, which are then transported to the liver; this leads to the production of small dense LDL (LDLsd) and decreased levels of HDL-c, which substantially influences the development of atherosclerosis [10].

Atherogenic indices could adequately reflect the clinical and metabolic interactions of lipid fractions [11]. The total cholesterol (TC)/HDL ratio is considered to have a high differentiating power for coronary heart disease in addition to a high predictive capacity [12]. The TG/HDL ratio has been shown to have a high association with the prevalence of metabolic syndrome and insulin resistance [13].

It has also been determined that the high TG/low HDL-C phenotype is associated with LDLsd particles that represent the most prevalent forms for ischemic cardiovascular disease; this ratio has been supported as a risk indicator for lipid atherogenesis, similar to the TC/HDL-C ratio [12].

Non-HDL cholesterol is defined as the sum of LDL-c, VLDL cholesterol, and remnant particle cholesterol. Non-HDL cholesterol can provide information on the balance between atherogenic and antiatherogenic lipoproteins represented by HDL-c. This is especially useful in common diseases such as diabetes mellitus, metabolic syndrome, and visceral obesity, where atherogenic dyslipidemia (increased triglycerides, decreased HDL-c, and increased LDLsd particles) is a feature. Therefore, non-HDL cholesterol may be a marker of residual lipid risk [14].

The present study was carried out on citizens of the city of Trujillo, since in a previous study a high prevalence of dyslipidemias and a high percentage of atherogenic indicators of cardiovascular risk levels were observed in people over 30 years of age [15]. However, it is not known how to predict these indicators in the population of Trujillo in a simpler and more direct way than the risk factors associated with CVD. Therefore, the objective was to determine predictive models for atherogenic risk, which were assessed using the indicators TC/HDL, triglycerides/HDL, LDL/HDL, and non-HDL cholesterol, which are based on dietary habits, healthy lifestyles, the presence of family history, and body mass index.

## 2. Materials and Methods

### 2.1. Type and Design of Research

The present study is a cross-sectional, non-experimental design with a predictive level according to the sex of the participants.

### 2.2. Population, Sample and Sampling

The population consisted of all the citizens of the city of Trujillo. To determine the sample size, the formula for an infinite population was used, considering a 6% error margin and 95% confidence level, resulting in 267 participants in the study [16].

Non-probabilistic convenience sampling was considered, with participants being former students of the Colegio Nacional San Juan, a national emblematic institution in the city of Trujillo. The director contacted the participants via email, text messages to their mobile phones, and/or Facebook, emphasising the invitation also to their families. The participants were registered by the researchers and the date of their assessment was arranged individually. The anthropometric evaluation, biochemical analysis, and application of lifestyle and family history questionnaires were carried out at the César Vallejo University in the Nutritional Evaluation Laboratories of the Professional School of Nutrition and the Research Laboratory of the Faculty of Health Sciences.

**Figure 1:**
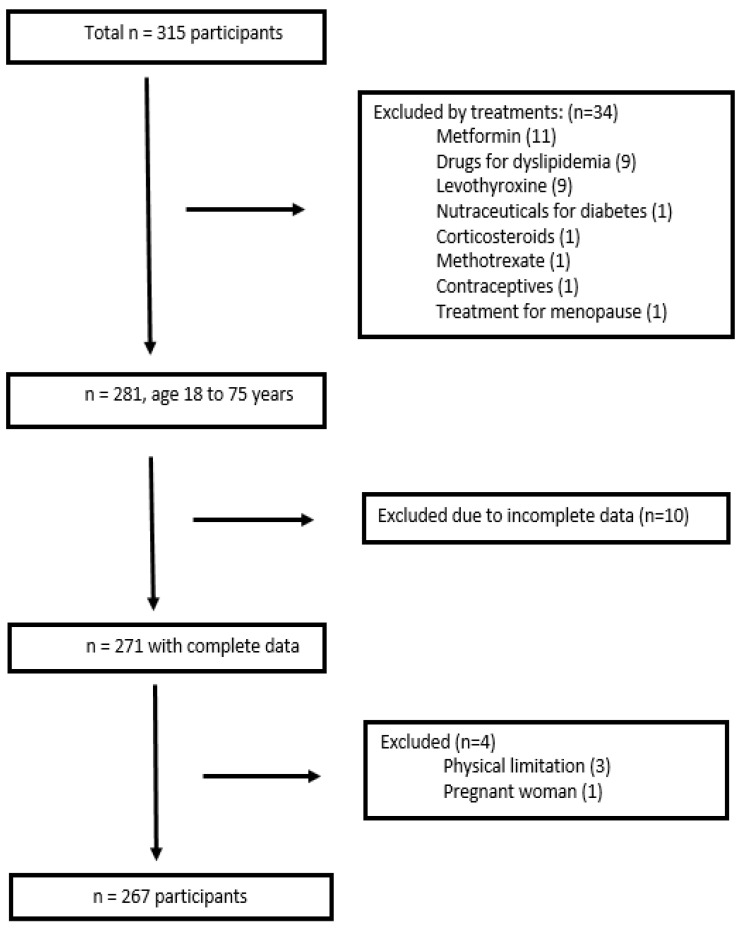
Flow diagram of subject inclusion and exclusion.

Persons over 18 years of age, healthy or with comorbidities, were included, with an initial number of 315 persons recruited. Those who consumed drugs for the treatment of dyslipidemias, metformin, levothyroxine, nutraceuticals for the treatment of various non-communicable diseases, corticosteroids, methotrexate, hormones for the treatment of menopause, or contraceptives were excluded. Also, those who presented incomplete data in the records of biochemical and anthropometric data, who did not fully complete the questionnaires, who showed the presence of any physical limitation, and pregnant women were excluded. Finally, 267 participants remained for data analysis, as summarized in Figure 1.

### 2.3. Evaluation of Baseline Characteristics

Participants were instructed to present themselves for analyses after a ten-hour fast. These analyses were conducted twice a week in the morning at 8 a.m., from September to December 2023, in the Nutritional Evaluation Laboratory of the School of Nutrition and the Research Laboratory of the Faculty of Health Sciences at César Vallejo University. For the lipid profile evaluation, cholesterol monitoring equipment (Mission^®^, Acon laboratories, San Diego, CA, USA) was used. Capillary blood was obtained from the index or middle finger. The finger was cleaned with 96° alcohol, and, once dry, the retractable lancing device was applied. The first drop of blood was wiped away, a slight pressure was applied to obtain a second drop of blood, and 35 μL was collected up to the fill line in the capillary transfer tube. This was then added to a test strip previously placed in the cholesterol meter. Normal values were considered to be TC < 200 mg/dL, triglycerides < 150 mg/dL, and LDL < 100 mg/dL; acceptable values of HDL in men and women were considered to be ≥40 mg/dL and ≥ 50 mg/dL, respectively [17].

For glycemia, blood from the same tube used for cholesterol was placed on a test strip inserted into a glucometer (Accu-Chek^®^ Performa Nano, Mannheim, Germany). A fasting glucose value of less than 100 mg/dL was considered normal [18]. Blood pressure was measured after a rest period of at least five minutes using a digital arm sphygmomanometer (Riester^®^ Ri-Champion N, Jungingen, Germany). For data interpretation, systolic blood pressure (SBP)/diastolic blood pressure (DBP) was considered optimal < 120/80 mmHg, normal from 120–129/80–84 mmHg, high from 130–139/85–89 mmHg, and hypertension ≥ 140/90 mmHg [19]. Abdominal circumference was measured with a metallic tape measure (Lufkin W606PM, Queretaro Mexico), and abdominal obesity was considered for waist circumference ≥ 94 cm in men and ≥88 cm in women [20]. Quantitative data for all these parameters were recorded on a data collection sheet.

### 2.4. Evaluation of Atherogenic Indicators

The atherogenic indices used for cardiovascular risk diagnosis were as follows: TC/HDL (Castelli Risk Index I), considering a value ≥ 4.5 for women and ≥5 for men as indicative of risk; TG/HDL-c greater than 3 as a risk for CVD; LDL/HDL (Castelli Risk Index II), considering low risk < 3 and high risk ≥ 3 [21].

Finally, non-HDL cholesterol was considered low risk < 130 mg/dL and high risk ≥ 130 mg/dL [14]. These data were recorded on a data collection sheet.

### 2.5. Dietary Habits

The questionnaire from the Senior Adult Food Quality Survey (ECAAM) was used, whose content was validated in a study by Durán et al. [22], using the calculation of Lawshe’s content validity ratio, with a value of 0.85. The instrument contains 21 questions, divided into two parts. The first 13 questions pertain to healthy eating habits related to breakfast, consumption of skimmed dairy products, fresh fruits, vegetables (portions equivalent to one plate), fish (fresh/frozen/canned, but not fried), legumes, oats/whole grain breads, consumption of homemade meals, dinner (meal + fruit and/or salad), water or liquids (herbal waters, fruit juices, tea, mate), meat/poultry, eggs, and number of meals per day. The second part consists of eight questions regarding unhealthy eating habits related to the consumption of sugary drinks or juices, alcoholic beverages, fried food, bakery products (cakes, pastries)/cookies, use of butter/margarine in preparations, fast food consumption, coffee consumption, and adding salt to meals before tasting them.

For the questions about healthy eating habits, the interviewee was given five response options per question, with values ranging from 1 point for ‘no consumption’ to 5 points for maximum consumption, depending on the food. For unhealthy eating habits, the response values were considered in reverse: 1 for maximum consumption and 5 for no consumption, except for question 21 regarding ‘adding salt’ which had three responses: always (1 point), occasionally (2 points), does not add (3 points). After adding all the response scores, their diet type was categorized as good (83–103 points) or poor (21–82 points).

### 2.6. Physical Activity

The International Physical Activity Questionnaire (IPAQ) short version, consisting of seven open-ended questions, was used. This allowed for the assessment of participants’ physical activity levels as low, moderate, or high according to the weekly frequency and duration of physical activity in minutes/day and the use of the metabolic equivalent of a task (MET) per minute, as well as the amount of time participants spent sitting each day [23]. This questionnaire demonstrated a reliability of 0.76; furthermore, a concordance of 0.67 (95% CI: 0.64–0.70) was found between the long and short versions [24], which is why it was considered for determining the prevalence of physical activity.

### 2.7. Alcohol and Tobacco Consumption

Alcohol consumers were defined as those who consumed alcoholic beverages at least once a fortnight, regardless of the amount, with the categories established as “alcohol consumer” and “non-alcohol consumer”. For tobacco consumption, “smoker” was defined as those participants who, at the time of the study, currently smoked or had quit smoking in the past month and consumed at least one cigarette per day or tobacco in any form. Two categories were established: “tobacco consumer” and “non-tobacco consumer” [25].

### 2.8. Hours of Sleep

The National Sleep Foundation recommends that adults sleep at least 7 h per day. Therefore, the following indicators were considered for evaluation in the present study: <7 h (short sleep); 7 to 9 h (recommended sleep); and 10 h or more (extended sleep). It has been reported that both short sleep duration (<7 h) and extended sleep duration (>9 h) are associated with a higher risk of morbidity and mortality, including cardiovascular and cerebrovascular diseases, obesity, diabetes, cancer, and depression [26].

### 2.9. Family History

Participants were asked if their parents and grandparents have or had diabetes mellitus, hypertension, dyslipidemia, or CVD [25].

### 2.10. Body Mass Index

Body mass index (BMI) was determined using the formula weight/height^2^, expressed in kg/m^2^ according to WHO criteria, with the following categories: underweight (<18.50 kg/m^2^); normal weight (18.50–24.99 kg/m^2^); overweight or pre-obesity (25.00–29.99 kg/m^2^); and obesity (≥30.00 kg/m^2^) [27]. Weight was assessed using an electronic floor scale (SECA 813, Hamburg, Germany) and height was assessed using a stadiometer (SECA 213, Hamburg, Germany).

### 2.11. Statistical Analysis

The data were transcribed from a data collection sheet (see Supplementary Material) into a Microsoft Excel spreadsheet and then exported to the statistical program SPSS version 26, where descriptive statistical calculations such as measures of central tendency (mean and standard deviation) were performed. In inferential statistics, the chi-square test was used to assess the association or differences between qualitative variables such as lifestyle and family history according to the gender of the participants. For quantitative variables like baseline characteristics by gender, the Mann-Whitney U test was used for independent samples [28]. Multivariate statistics were also employed using binary logistic regression [29] to determine models for factors associated with atherogenic risk in participants generally and by sex, with a 95% confidence interval and a significance level of 0.05. In addition to including the nominal variable sex in the predictive model analysis, age was considered a quantitative variable since the lipid profile varies with physiological changes. Variables entering the study models were selected based on *p* < 0.05 significance, the OR, and its confidence intervals. When the odds ratio (OR) is greater than 1 and its confidence intervals are also greater, the variable constitutes a risk factor, and when the OR and its confidence intervals are less than 1, the variable constitutes a protective factor.

### 2.12. Ethical Aspects

The present work has been approved by the Ethics Committee of the Professional School of Nutrition of the Universidad César Vallejo with report PI-CEI-NUTRICIÓN-2023-003. The ethical principles of beneficence, non-maleficence, autonomy, and justice, contained in the declaration of Helsinki and the code of ethics of the Universidad César Vallejo, were considered. Each participant was provided with detailed information about the objectives of the study, the basic methods and/or protocols of analysis to be implemented, and the possible discomforts during the process. This ensured that participants had a full understanding of the information and could decide to accept or withdraw from participation in the research.

## 3. Results

### 3.1. Baseline Characteristics of the Participants

In Table 1, it can be observed that age, diastolic pressure, and triglyceride concentration do not differ significantly between males and females. In the rest of the characteristics, males have higher averages in abdominal circumference, blood glucose, systolic pressure, TC/HDL, TG/HDL, and LDL/HDL, all of which exceed the threshold values for cardiovascular risk, except for systolic pressure, where the average value is within the normal range. Cholesterol, LDL, and non-HDL cholesterol are higher in females and exceed their normal thresholds, indicating a higher cardiovascular risk. The average HDL concentration is higher in women than in men; however, in both genders, it is below the normal values of 40 and 50 mg/dL for men and women, respectively.

### 3.2. Evaluation of Lifestyle and BMI

In Table 2, regarding lifestyle, the majority of participants have poor dietary habits, low physical activity, sleep less than 7 h, and do not consume tobacco or alcohol.

In addition, within the male group, 92.5% (99/107) have poor dietary habits, a significantly higher proportion (*p* < 0.05) than that observed in women, at 81.9% (131/160).

In relation to the total number of participants, 17.6% of men have high physical activity, while 25.5% have low physical activity, highlighting this difference between the two groups (*p* < 0.05). It is also observed that a higher proportion of women get less than 7 h of sleep compared to men (*p* < 0.05). Although a higher proportion of men than women use tobacco, this difference is not significant (*p* > 0.05). As for alcohol consumption, it is low, but predominantly higher in men than in women (*p* < 0.05).

Regarding BMI, in the male group, overweight represents 50.5% (54/107) and obesity represents 37.4% (40/107) of the population, significantly higher proportions compared to the female group, where they represent 39.4% (63/169) and 26.3% (42/160) of the population, respectively.

### 3.3. Family History of Cardiovascular Risk

Table 3 shows that there is only a relationship between the history of CVD in the mother and the gender of the participants (*p* < 0.05). This family history occurs significantly within the female gender, with a proportion of 58.8% (94/160), representing 35.2% of the total participants.

### 3.4. Prediction for Atherogenic Risk Determined by TC/HDL

Table 4 shows the general binary logistic regression model for TC/HDL in residents of the city of Trujillo, including the variables of grandfather’s history, BMI, and sex. This indicates that a person whose grandfather has a history of CVD is approximately twice as likely to be diagnosed with atherogenic risk due to elevated TC/HDL (OR 1.898 [1.036; 3.478], *p* = 0.038). Regarding BMI, normal weight and overweight are better than obesity (reference category) and are protective factors for the diagnosis of elevated TC/HDL. For the gender variable, the model shows that a diagnosis of elevated TC/HDL is about twice as likely to be associated with atherogenic risk in women (OR 1.814 [1.037; 3.172], *p* = 0.038).

The Hosmer–Lemeshow test indicates that the model fits the data adequately. The R^2^ values of Cox, Snell, and Nagelkerke indicate that the model explains 15 to 20% of the behavior of the TC/HDL variable; however, the prediction percentage is 66%, which, being greater than 50%, is considered an acceptable model. Additionally, the area under the receiver operating characteristic (ROC) curve, abbreviated as AUC, indicates that there is a 72% probability that the model correctly classifies healthy and sick individuals. The model is established as follows:

(1) $$p(Y)=\frac{1}{{1+e}^{-(0.641X1-1.871X2-1.152X3+0.595X4)}}$$

where Y is diagnosis of elevated TC/HDL, X1 is history of CVD in the grandparent, X2 is BMI (normal), X3 is BMI (overweight), and X4 is sex

In the binary logistic regression model for women, grandfather’s history of CVD is included as a risk factor with an approximate OR of 2.3 (OR 2.312 [1.062; 5.031], *p* = 0.035) and normal BMI is included as a protective factor against elevated TC/HDL. The R^2^ values of Cox, Snell, and Nagelkerke indicate that the model explains only 10 to 14% of the behavior of the TC/HDL variable, with an acceptable prognosis of 64%. The AUC indicates a 68% probability that the model correctly classifies healthy and sick individuals.

For the binary logistic regression model in men, only BMI in its normal and overweight categories enters as a protective factor. The model fits the data adequately; the R^2^ values of Cox, Snell, and Nagelkerke indicate that the model explains 15 to 21% of the behavior of the TC/HDL variable, with an acceptable prognosis of 73%. The AUC indicates a 71% probability that the model correctly classifies sick and healthy individuals.

### 3.5. Prediction for Atherogenic Risk Determined by TG/HDL

Table 5 shows the general binary logistic regression model for TG/HDL in residents of the city of Trujillo, including the variables of low physical activity, alcohol consumption, BMI, and sex. The BMI variable in its normal and overweight categories is a protective factor against the diagnosis of elevated TG/HDL, with obesity as the reference category. Therefore, having a normal or overweight BMI is better than being obese to reduce the likelihood of being diagnosed with elevated TG/HDL. Low physical activity, alcohol consumption, and male gender are risk factors for elevated TG/HDL.

This indicates that there is approximately 2.4 times greater probability for a person with low physical activity to present atherogenic risk due to elevated TG/HDL compared to a person who does engage in physical activity (OR 2.359 [1.150; 4.838], *p* = 0.019). Alcohol consumption increases the probability of a person having atherogenic risk due to elevated TG/HDL by 3 times (OR 3.077 [1.072; 8.832], *p* = 0.037). However, in males, the probability of presenting atherogenic risk is approximately 4 times higher compared to females (OR 3.902 [2.092; 7.276], *p* < 0.001).

The Hosmer–Lemeshow test indicates that the model is a good fit to the data. The Cox, Snell, and Nagelkerke R^2^ values indicate that the model explains 23 to 31% of the behaviour of the TG/HDL variable. The percentage of prediction is 70%, which, being greater than 50%, is considered an acceptable model. In addition, the AUC indicates a 78% probability that the model correctly classifies healthy and sick individuals. The model is established as follows:

(2) $$p(Y)=\frac{1}{{1+e}^{-(0.858X1.+1.124X2-2.371X3+-1.232X4+1.361X5)}}$$

where Y is diagnosis of elevated TG/HDL, X1 is low physical activity, X2 is alcohol consumption, X3 is BMI (normal), X4 is BMI (overweight), and X5 is sex.

For the binary logistic regression model for women, only normal BMI is included as a protective factor. The R^2^ values of Cox, Snell, and Nagelkerke indicate that the model explains only 10 to 14% of the behavior of the TG/HDL variable, with an acceptable prediction of 66%. The AUC indicates a 68% probability that the model correctly classifies healthy and sick individuals.

For the binary logistic regression model in men, the variables of alcohol consumption and BMI in the normal and overweight categories are included. Additionally, in the male group, the probability of presenting atherogenic risk is approximately 7.5 times higher in those who consume alcohol compared to non-consumers (OR 7.494 [1.742; 32.247], *p* = 0.007). The R^2^ values of Cox, Snell, and Nagelkerke indicate that the model explains 26 to 36% of the behavior of the TG/HDL variable, with an acceptable prediction of 78%. The AUC curve indicates a 78% probability that the model correctly classifies healthy and sick individuals.

### 3.6. Prediction for Atherogenic Risk Determined by LDL/HDL

Table 6 shows the general binary logistic regression model for LDL/HDL in residents of the city of Trujillo, incorporating the BMI variable in its normal weight and overweight categories as a protective factor against the diagnosis of elevated LDL/HDL and female gender as a risk factor. In women, there is approximately 4 times the probability of presenting atherogenic risk compared to men.

The R^2^ values of Cox, Snell, and Nagelkerke indicate that the model explains 15 to 20% of the behavior of the LDL/HDL variable. The prediction percentage is 68%, which, being greater than 50%, is considered an acceptable model. Additionally, the AUC indicates a 72% probability that the model correctly classifies healthy and sick individuals. The model is established as follows:

(3) $$p(Y)=\frac{1}{{1+e}^{-(1.147-1.628X1-1.218X2-1.085X3)}}$$

where Y is diagnosis of elevated LDL/HDL, X1 is BMI (normal), X2 is BMI (overweight), and X3 is female sex.

For the binary logistic regression model for women, only normal BMI is included as a protective factor. The R^2^ values of Cox, Snell, and Nagelkerke indicate that the model explains only 8 to 11% of the behavior of the LDL/HDL variable, with an acceptable prediction of 62.5%. The AUC indicates a 66% probability that the model correctly classifies healthy and sick individuals.

For the binary logistic regression model in men, BMI in its normal and overweight categories is included. The R^2^ values of Cox, Snell, and Nagelkerke indicate that the model explains 17 to 26% of the behavior of the LDL/HDL variable, with an acceptable prediction of 79.4%. This model has a better area under the curve than the previous models, indicating a 73% probability of correctly classifying sick and healthy individuals.

### 3.7. Prediction for Atherogenic Risk Determined by Non-HDL Cholesterol

Table 7 shows the binary logistic regression model for non-HDL cholesterol in residents of the city of Trujillo, where, similar to the previous models, the variable BMI in its normal and overweight categories is included as a protective factor against the diagnosis of elevated non-HDL cholesterol. Age is also included as a risk factor for the development of elevated non-HDL cholesterol, indicating that with increasing age there is a 1.030 increased probability of having elevated non-HDL cholesterol.

The R^2^ values of Cox, Snell, and Nagelkerke indicate that the model explains 9 to 13% of the behavior of the non-HDL cholesterol variable; however, the prediction percentage is 74%, which, being greater than 50%, is considered an acceptable model. Additionally, the AUC indicates a 69% probability that the model correctly classifies healthy and sick individuals. The model is established as follows:

(4) $$p(Y)=\frac{1}{{1+e}^{-(-1.727X1-1.394X2+0.030X2)}}$$

where Y is diagnosis of elevated non-HDL cholesterol, X1 is BMI (normal), X2 is BMI (overweight), and X3 is age.

For the binary logistic regression model for women, normal BMI is included as a protective factor and age is included as a risk factor. The R2 values of Cox, Snell, and Nagelkerke indicate that the model explains only 12 to 17% of the behavior of the non-HDL cholesterol variable, with an acceptable prediction of 77%. The AUC indicates a 73% probability that the model correctly classifies healthy and sick individuals.

For the binary logistic regression model in men, only BMI in its normal and overweight categories is included. The model fits the data adequately, and the R^2^ values of Cox, Snell, and Nagelkerke indicate that the model explains 10 to 15% of the behavior of the non-HDL cholesterol variable, with an acceptable prediction of 73%. According to the AUC value, the model has a 68% probability of correctly classifying sick and healthy individuals.

## 4. Discussion

The baseline characteristics by gender shown in Table 1 vary compared to those found in other studies, such as the study by Mohammedsaeed [30] in Saudi Arabia. In this study, both blood glucose and lipid profile parameters show that women have higher values than men, and both are at risk levels, except for triglycerides, where the concentration is higher in men but within normal levels. In research conducted in some Asian countries [12,31], the lipid profile is adequate, with the average HDL concentration exceeding 40 mg/dL in men and 50 mg/dL in women; additionally, they also have abdominal circumference, systolic and diastolic blood pressure, and blood glucose within normal values. In Peru, a study with similar results was reported by Mayta and Palomino [32] in Lima for a group of workers, where the average triglyceride concentration among men and women is slightly above 150 mg/dL and the total cholesterol concentration is slightly above 190 mg/dL; however, it differs from the average of 120 mg/dL for LDL, as in Trujillo, the average among residents exceeds 130 mg/dL. Additionally, in this study, elevated TC/HDL and TG/HDL indices were found in 40% and 60% of participants, respectively. In Chachapoyas, a place in the Amazon highlands, similar trends are observed, although hypertriglyceridemia is more prominent in men, with a value close to 160 mg/dL compared to an average of 125 mg/dL in women, and 73% of participants have central obesity and an overweight BMI [33].

The difference in the lipid profile with higher total cholesterol and LDL concentration in women compared to men is due to an increase in cholesterol levels in women, particularly after menopause and the corresponding reduction in estrogen [34,35], coinciding with the average age of the participants, which is similar to that of men. Despite women having higher HDL values compared to men, this cannot compensate for the imbalance imposed by the increase in LDL concentration established during hormonal changes in postmenopause, as the average HDL in women is below 50 mg/dL, its threshold value.

The elevated values of atherogenic indices found in the study participants indicate the need to consider public prevention strategies for this disease at the national level. It is known that the consumption of ultra-processed foods is an influential factor that, on average, over 5 to 7 years, can lead to the observed dyslipidemias and therefore high values of atherogenic indices [36].

The lifestyles of the study participants have also been observed in Zhang et al. [37], where both men and women engaged in physical activity less than once a week and men had higher tobacco consumption.

The prevalence of alcohol consumption in the city of Trujillo is slightly higher than the national level found by Caira-Chuquineyra et al. [38] in 2022, with men being the highest consumers.

Additionally, the trend of higher prevalence of overweight and obesity in women compared to men has been previously observed in a study conducted in three countries, Mexico, Colombia, and Peru, based on data recorded between 2005 and 2013 [39]. Three quarters of the participants being overweight or obese is related to the high percentage of people with poor dietary habits, which appears to be an increasing issue observed in a previous study by Agurto et al. [40] in Peru. They associated weight gain in 40% of participants in Lima with poor dietary habits during the pandemic, as well as with poor sleep quality of less than 7 h and reduced physical activity, similar to that observed in other Latin American contexts [41,42] and similar to that found in the city of Trujillo.

The association of sleep with obesity and even dyslipidemias has been demonstrated in various studies [43,44,45]. These pathologies were more prevalent in the present study, likely due to chronic conflict between the circadian rhythm and time constraints for rest depending on the activities that people develop [46].

Furthermore, the excessive consumption of carbohydrates characteristic of the Peruvian population leads to overweight and obesity, nutritional states that have increased compared to even national data in 2022, where the prevalence of overweight was 37.5% and obesity 25.6% of the adult population, more prevalent in women [47], with a similar trend observed in the city of Trujillo.

Regarding family history, there are reports of a family history of coronary artery disease in patients who present with this condition, as in the study by Faggiano et al. [48], which found a prevalence of 32.8% of this genetic history through a multicenter hereditary survey, where 88% of the patients had dyslipidemia. As shown in Table 3, family history with cardiovascular risk ranges from 25.1% to 44.9% in a population with a high index of dyslipidemia, demonstrated by elevated values in all atherogenic indices and low HDL levels, indicating a significant prevalence closely related to familial dyslipidemia. A similar prevalence was determined in the study by Mehta et al. [49], with 44% of atherosclerosis risk due to family history, predominantly in women.

The logistic model shown in Table 4, which explains elevated TC/HDL values, indicates the importance of considering family history knowledge. In a study by Barbalho et al. [50], family history was found to be related to the TC/HDL index in Brazilian patients who had undergone coronary arteriography. However, the study did not specify if this was due to parents and/or grandparents. In the present study, a history of CVD in the grandfather was found to be relevant information for diagnosing atherogenesis.

Regarding BMI, this indicator was involved in a binary regression model determined by González Jaime et al. [51], along with other anthropometric indicators not evaluated in the present study, such as waist circumference, waist-to-hip ratio, waist-to-height ratio, neck circumference, visceral fat, and fat percentage, which were also predictor variables for atherogenic risk evaluated with the Castelli index.

Similarly, in the CT/HDL prediction model, the probability of this indicator is higher in women, which appears to be more related to decreases in estrogen levels. This is consistent with the high average total cholesterol value in this group of 210.72 ± 51.54 mg/dL above the limit and higher than in men, whose average HDL concentration is below the threshold of 50 mg/dL observed in Table 1. This leads to a higher likelihood of the TC/HDL ratio exceeding 4.5 and presenting atherogenic risk diagnosis in the majority of women. Therefore, weight loss is a well-established method to achieve higher HDL concentrations [52], which thus establishes lower TC/HDL values, below the limit for both men and women.

Kligs et al. [53] state that the high risk of metabolic syndrome is transmitted from parents to children and that this intergenerational transmission of metabolic syndrome is similar for sons and daughters, regardless of socioeconomic status and health behaviours of the children. The present study determined heritability from the grandfather’s cardiovascular disease history in the predictive model for atherogenic risk diagnosed by CT/HDL in female participants, with the probability of having it being approximately twice as high. Therefore, it is necessary to investigate possible genes that may be present from the second degree of consanguinity.

The BMI in obesity, low physical activity, alcohol consumption, and male gender in the general logistic regression model for predicting the TG/HDL ratio shown in Table 5 reach an approximate probability of 93% in men and 76.92% in women for this indicator to be elevated. This differs from a study by Moriyama et al. [31], which found that the TG/HDL ratio, in addition to being associated with physical activity, was also associated with smoking, but not with alcohol consumption, as was found in the present study. The TG/HDL is highly related to small, dense low-density lipoprotein cholesterol (sdLDL) and oxidized LDL, particles involved in the development of atherosclerosis [31] and also in long-term mortality in high-risk patients [54], pathophysiological aspects that are increased in obesity. Therefore, as in the other model, obesity BMI should be reduced, and for this, physical activity needs to be increased in both sexes.

The higher risk of elevated TG/HDL in men, as evidenced by the approximate OR of 4, appears to be related to abdominal visceral fat, which is more pronounced in men with android obesity (apple shape) compared to women with a gynoid fat distribution. Additionally, men generally consume a greater amount of dietary fat due to their higher energy intake, producing larger and more numerous chylomicrons than women and thus greater storage in abdominal visceral fat [55].

Furthermore, the decrease in testosterone as men age leads to a reduction in skeletal muscle mass and an increase in visceral fat, which results in a greater influx of free fatty acids from visceral fat to the liver, inducing hepatic insulin resistance and reducing the synthesis of sex hormone-binding globulin (SHBG). Additionally, low testosterone levels, along with lower muscle mass and higher fat mass, inversely correlate with the expression of oxidative phosphorylation genes in mitochondria, crucial for insulin resistance. The decrease in SHBG might not suppress hepatic lipogenesis, thus exacerbating insulin resistance [56].

In the present study, alcohol consumption was found to be directly associated with the TG/HDL index in both the general model (OR 3.902 [2.092; 7.276], *p* < 0.001) and in men for the prediction of atherogenic risk (OR 7.494 [1.742; 32.247], *p* = 0.007). This differs from a study by Shimomura and Wakabayashi [57], who found an inverse relationship between light to moderate alcohol consumption and high TG/HDL values. The relationship between alcohol consumption and body weight is usually more pronounced in men than in women, mainly due to the amount and type of alcohol consumed. Moreover, men tend to prefer beer, which is high in carbohydrates and provides more energy per standard drink compared to wine [58]. This corresponds to the higher proportion of alcohol consumption observed in men in the city of Trujillo shown in Table 2. Additionally, there is a higher consumption of beer, which is also in line with the preferences of Latin Americans [59].

High alcohol consumption is closely linked to an increase in visceral abdominal fat. This is because alcohol, with its high energy content (7.1 kcal/g), contributes to weight gain by adding to the total daily caloric intake. Furthermore, alcohol can promote food consumption by inhibiting satiety-related hormones such as leptin and glucagon-like peptide-1 [60].

Additionally, the elevated TG and low HDL indices clearly lead to the generation of the atherogenesis process from the moment TG-rich very low-density lipoprotein (VLDL) is secreted by the liver into the bloodstream. Triglycerides are transferred to LDL particles through the cholesterol ester transfer protein (CETP), allowing hepatic lipases to hydrolyze triglycerides, forming smaller LDL particles [61].

Additionally, Areum and Saeron [62] associated gender and BMI values greater than 30 kg/m^2^ with elevated LDL/HDL. Considering this BMI cut-off point, the equation shown in Table 6 predicts approximately a 51.55% probability of elevated LDL/HDL if the person is obese and female. This differs from Sun et al. [63], who found a relationship between LDL/HDL and various BMI levels, with a cut-off point of 2.5 for LDL/HDL, where 87% of the obese group fell. The general LDL/HDL model in Table 6 should be improved, as there is a higher concentration of cholesterol in VLDL in people with high TG levels. Thus, the LDL/HDL ratio may underestimate the extent of dyslipidemia in these patients [64], which was not considered. The inclusion of the female gender in this model is mainly related to the previously mentioned decrease in estrogen concentration.

In the final model presented in Table 7, a high prediction of non-HDL cholesterol is shown for a value above 130 mg/dL in an obese person with an average age of 50 years, close to the participants’ average age, with an approximately 82% probability; compared to 71% for a 30-year-old, the prediction of elevated non-HDL cholesterol remains high. If a person with low BMI and this nutritional status is 50 years old, the probability is 44%. This is corroborated by the quantitative results for this indicator shown in Table 1, where the averages for both men and women exceed the acceptable level for non-HDL cholesterol, as they are 151.26 ± 37.35 mg/dL and 164.33 ± 51.01 mg/dL, respectively. This requires evaluating other metabolic aspects that may affect the increase in these particles, possibly involving an enzyme such as CETP. This was demonstrated by Coniglio et al. [65] in patients at risk of type 2 diabetes mellitus and those with metabolic syndrome, where high levels of non-HDL cholesterol predict higher CETP activity, which is also involved in decreasing HDL levels and antiatherogenic functions, possibly involving a gene mutation for this enzyme and associated with age [66].

Non-HDL cholesterol is a simpler, more convenient, and predictive measure than LDL cholesterol. Although less frequently used, it is especially useful in the context of mild to moderate hypertriglyceridemia and often accompanies cardiometabolic disorders such as diabetes, obesity, and metabolic syndrome. Unlike LDL cholesterol, non-HDL cholesterol does not require complex calculations, as it is obtained by subtracting HDL cholesterol from total cholesterol. This makes it more reliable, as it does not depend on triglycerides for its calculation as LDL does, and triglycerides can vary in individuals due to diet, potentially biasing LDL values. In summary, non-HDL cholesterol is a solid tool for assessing cardiovascular risk [67].

One limitation of this study is the cross-sectional data collection for each variable, meaning the observed regression models did not allow for the inclusion of more variables over time with participant follow-up. This could include poor eating habits and/or less than 7 h of sleep, which were negative aspects with higher prevalence. Despite the small, non-probabilistic sample size, developing it with participants from a prominent city school where former students come from various districts provides an approximation of the current reality. However, it is important to expand the sample nationally and regionally to identify predictive models of atherogenic risk that generalize results to contribute to national healthy eating policies.

Another limitation in determining the predictive models was the exclusion of metabolic markers such as adipocytokines, including leptin and adiponectin, among other inflammatory markers that also influence atherogenic risk [68], due to time and budget constraints. Another limitation is the existence of genetic variations in certain genes involved in lipoprotein metabolism that could be related to atherogenesis, such as the FTO gene, which is associated with obesity, insulin resistance, and BMI changes, respectively [69]. It is therefore necessary to carry out longitudinal studies that take these factors into account in order to provide greater support for the models found. These studies will also enable the discovery of interactions between lifestyle, dietary composition, metabolic markers, and the genes involved, which will help to understand atherogenic risk from a nutrigenetic and epigenetic perspective and to detect it early both in individualised clinical settings in patients and in population settings, thus contributing to preventive public health.

A strength of this study is that it is the first of its kind conducted in the city of Trujillo and in Peru, where we determined the prediction of atherogenic risk. It will also allow interesting future studies on the genetic aspect, as it found a notable relevance of family history of CVD in grandfathers in predicting atherogenic risk using the Castelli index.

Public health policies should focus on the involvement of multidisciplinary teams, especially nutritionists, from the first level of care (prevention), to carry out nutritional education activities at different ages. Public health should aim to control and regulate the indiscriminate consumption of unhealthy foods (fast food), which will have fatal consequences for the population in the near future. For this reason, the Dietary Guidelines for the Peruvian Population help to guide the general public on proper diet and nutrition, promoting good eating habits, such as the practice of a properly proportioned plate (half a plate of vegetables, a quarter of cereals and a quarter of animal products), reducing processed and ultra-processed foods, avoiding sugary drinks and foods, and encouraging physical activity for at least 30 min a day and reducing alcohol consumption, especially among men, for a full life free of cardiovascular risk diseases [70].

## 5. Conclusions

It is concluded that a family history of CVD from the grandparent and female sex are risk factors in the atherogenic risk prediction model determined by the total cholesterol/HDL index. Regarding the atherogenic risk determined by TG/HDL, this indicator presents a predictive model in which low physical activity, obesity BMI, sex, and alcohol consumption (the latter more linked in men) are considered risk factors. In the atherogenic risk prediction models determined by LDL/HDL and non-HDL cholesterol, female sex and age are the main risk factors, respectively, in addition to BMI. Of all the models, the one that best predicts atherogenic risk is the one for TG/HDL, as it involves more entered variables and presents a larger area under the curve, while also considering a family history of CVD, especially from the grandparent, and age. It is necessary to add more metabolic and genetic markers to establish a better-directed nutritional treatment, whether individual or population-wide.

## Figures and Tables

**Table 1:** Baseline characteristics by gender in residents of the city of Trujillo.

Characteristics	Gender			Sig.
	Male	Female	Total	
	(*n* = 107)	(*n* = 160)	(*n* = 267)	
Age (years)	47.54 ± 10.83	47.09 ± 11.71	47.27 ± 11.34	0.66
Abdominal circumference (cm)	97.76 ± 11.47	87.21 ± 10.28	91.44 ± 11.94	0.00 *
Blood glucose (mg/dL)	103.14 ± 16.35	101.57 ± 20.7	102.20 ± 19.05	0.01 *
Systolic BP (mmHg)	117.43 ± 14.85	111.44 ± 17.23	113.83 ± 16.56	0.00 *
Diastolic BP (mmHg)	75.37 ± 10.04	72.94 ± 10.96	73.92 ± 10.65	0.06
Total cholesterol (mg/dL)	184.35 ± 38.03	210.72 ± 51.54	200.16 ± 48.29	0.00 *
Triglycerides (mg/dL)	162.17 ± 103.65	141.95 ± 87.02	150.05 ± 94.37	0.06
HDL (mg/dL)	33.10 ± 11.42	46.39 ± 14.75	41.07 ± 14.98	0.00 *
LDL (mg/dL)	128.26 ± 85.76	137.85 ± 43.99	133.99 ± 64.18	0.02 *
Total cholesterol/HDL	6.10 ± 2.24	4.94 ± 1.90	5.41 ± 2.12	0.00 *
TG/HDL	5.86 ± 5	3.64 ± 3.31	4.53 ± 4.21	0.00 *
LDL/HDL	3.98 ± 1.66	3.24 ± 1.47	3.54 ± 1.59	0.00 *
Non-HDL cholesterol (mg/dL)	151.26 ± 37.35	164.33 ± 51.01	159.09 ± 46.40	0.04 *

Note: Mann–Whitney U test was used; * *p* < 0.05 is significant.

**Table 2:** Lifestyles and BMI by sex in residents of the city of Trujillo.

Lifestyles and BMI	Gender				Total		
	Male	%	Female	%		%	Sig.
Eating habits							
Bad	99	37.1%	131	49.1%	230	86.1%	0.01 *
Good	8	3.0%	29	10.9%	37	13.9%	
Physical activity							
Low	26	9.7%	68	25.5%	94	35.2%	0.00 *
Moderate	34	12.7%	51	19.1%	85	31.8%	
High	47	17.6%	41	15.4%	88	33.0%	
Hours of sleep							
Less than 7	60	22.5%	112	41.9%	172	64.4%	0.01 *
From 7 to 9	47	17.6%	44	16.5%	91	34.1%	
Older than 9	0	0.0%	4	1.5%	4	1.5%	
Tobacco use							
Yes	14	5.2%	10	3.7%	24	9.0%	0.06
No	93	34.8%	150	56.2%	243	91.0%	
Alcohol consumption							
Yes	17	6.4%	5	1.9%	22	8.2%	0.00 *
No	90	33.7%	155	58.1%	245	91.8%	
Body mass index							
Underweigt	1	0.4%	2	0.7%	3	1.1%	0.00 *
Normal	12	4.5%	53	19.9%	65	24.3%	
Overweight	54	20.2%	63	23.6%	117	43.8%	
Obesity	40	15.0%	42	15.7%	82	30.7%	
Total	107	40.1%	160	59.9%	267	100.0%	

Note: Chi-square test of association was used. * *p* < 0.05 is significant.

**Table 3:** Family history of cardiovascular risk by gender in residents of the city of Trujillo.

Family History Cardiovascular Risk	Gender				Total		
	Male	%	Female	%		%	Sig.
Father’s background							
Yes	51	19.1%	69	25.8%	120	44.9%	0.47
No	56	21.0%	91	34.1%	147	55.1%	
Mother’s background							
Yes	48	18.0%	94	35.2%	142	53.2%	0.03 *
No	59	22.1%	66	24.7%	125	46.8%	
Grandfather’s background							
Yes	25	9.4%	42	15.7%	67	25.1%	0.59
No	82	30.7%	118	44.2%	200	74.9%	
Grandmother’s background							
Yes	26	9.7%	55	20.6%	81	30.3%	0.08
No	81	30.3%	105	39.3%	186	69.7%	
Total	107	40.1%	160	59.9	267	100%	

Note: Chi-square test of association was used. * *p* < 0.05 is significant.

**Table 4:** Binary logistic regression model for TC/HDL in residents of the city of Trujillo.

General Model	Variables in the Equation						
	B	Error Estándar	Wald	Sig.	OR	95% C.I. Para OR	
						Lower	Upper
Grandfather’s background	0.641	0.309	4.305	0.038	1.898	1.036	3.478
BMI			23.780	0.000			
BMI (normal)	−1.871	0.390	22.981	0.000	0.154	0.072	0.331
BMI (overweight)	−1.152	0.340	11.444	0.001	0.316	0.162	0.616
Sex (female)	0.595	0.285	4.355	0.037	1.814	1.037	3.172
Constant	0.668	0.378	3.122	0.077	1.950		
Model summary: Predicted overall percentage: 66%							
Hosmer–Lemeshow test: Chi-square: 5.215 (*p* > 0.05)							
Cox–Snell R-squared: 0.15, Nagelkerke R-squared: 0.20							
Area under the curve: 0.72; CI: 0.66–0.78 (*p* < 0.05)							
Model for women							
Grandfather’s background	0.838	0.397	4.461	0.035	2.312	1.062	5.031
BMI			12.073	0.007			
BMI (normal)	−1.557	0.454	11.784	0.001	0.211	0.087	0.513
Constant	0.236	0.468	0.255	0.614	1.266		
Model summary: Predicted overall percentage: 64%							
Hosmer–Lemeshow test: Chi-square: 0.67 (*p* > 0.05)							
Cox–Snell R-squared: 0.10, Nagelkerke R-squared: 0.14							
Area under the curve: 0.68; CI: 0.596–0.764 (*p* < 0.05)							
Model for men							
BMI			11.562	0.009			
BMI (normal)	−2.534	0.788	10.344	0.001	0.079	0.017	0.372
BMI (overweight)	−1.667	0.598	7.776	0.005	0.189	0.059	0.609
Constant	2.197	0.527	17.380	0.000	9.000		
Model summary: Predicted overall percentage: 73%							
Hosmer–Lemeshow test: Chi-square: 0.00 (*p* > 0.05)							
Cox–Snell R-squared: 0.15, Nagelkerke R-squared: 0.21							
Area under the curve: 0.71; CI: 0.61–0.82 (*p* < 0.05)							

Note: SPSS version 27, Wald forward method.

**Table 5:** Binary logistic regression model for TG/HDL in residents of the city of Trujillo.

General Model	B	Standard Error	Wald	Next.	OR	95% CI for OR	
						Lower	Upper
Physical activity			6.180	0.046			
Physical activity (low)	0.858	0.366	5.484	0.019	2.359	1.150	4.838
Alcohol (yes)	1.124	0.538	4.364	0.037	3.077	1.072	8.832
BMI			32.378	0.000			
BMI (normal)	−2.371	0.424	31.271	0.000	0.093	0.041	0.214
BMI (overweight)	−1.232	0.339	13.224	0.000	0.292	0.150	0.567
Gender (male)	1.361	0.318	18.332	0.000	3.902	2.092	7.276
Constant	−0.778	0.609	1.630	0.202	0.459		
Model summary: Predicted overall percentage: 70%							
Hosmer–Lemeshow test: Chi Square: 10.50 (*p* > 0.05)							
Cox–Snell R-squared: 0.23, Nagelkerke R-squared: 0.31							
Area under the curve: 0.78, CI: 0.724–0.833 (*p* < 0.05)							
Model for women							
BMI			15.450	0.001			
BMI (normal)	−1.844	0.471	15.316	0.000	0.158	0.063	0.398
BMI (overweight)	−0.738	0.405	3.319	0.068	0.478	0.216	1.058
Constant	0.386	0.314	1.505	0.220	1.471		
Model summary: Predicted overall percentage: 66%							
Hosmer–Lemeshow test: Chi Square: 0.00 (*p* > 0.05)							
Cox–Snell R-squared: 0.10, Nagelkerke R-squared: 0.14							
Area under the curve: 0.68, CI: 0.596–0.764 (*p* < 0.05)							
Model for men							
Alcohol (yes)	2.014	0.745	7.318	0.007	7.494	1.742	32.247
BMI			15.267	0.002			
BMI (normal)	−3.554	0.940	14.288	0.000	0.029	0.005	0.181
BMI (overweight)	−2.184	0.709	9.504	0.002	0.113	0.028	0.451
Constant	0.887	0.665	1.782	0.182	2.429		
Model summary: Predicted overall percentage: 78%							
Hosmer–Lemeshow test: Chi Square: 0.272 (*p* > 0.05)							
Cox–Snell R-squared: 0.26, Nagelkerke R-squared: 0.36							
Area under the curve: 0.78, CI: 0.69–0.876 (*p* < 0.05)							

Note: SPSS version 27, Wald forward method.

**Table 6:** Binary logistic regression model for LDL/HDL in residents of the city of Trujillo.

General Model	B	Standard Error	Wald	Next.	OR	95% CI for OR	
						Lower	Upper
BMI			18.924	0.000			
BMI (normal)	−1.628	0.396	16.937	0.000	0.196	0.090	0.426
BMI (overweight)	−1.218	0.358	11.580	0.001	0.296	0.147	0.597
Sex(female)	1.085	0.300	13.087	0.000	2.959	1.644	5.326
Constant	1.147	0.313	13.395	0.000	3.149		
Model summary: Predicted overall percentage: 68%							
Hosmer–Lemeshow test: Chi-square: 5.215 (*p* > 0.05)							
Cox–Snell R-squared: 0.15, Nagelkerke R-squared: 0.20							
Area under the curve: 0.72; CI: 0.653–0.776 (*p* < 0.05)							
Model for women							
BMI			8.534	0.036			
BMI (normal)	−1.272	0.442	8.266	0.004	0.280	0.118	0.667
BMI (overweight)	−0.605	0.426	2.020	0.155	0.546	0.237	1.258
Constant	0.182	0.459	0.156	0.692	1.199		
Model summary: Predicted overall percentage: 62.5%							
Hosmer–Lemeshow test: Chi-square: 0.06 (*p* > 0.05)							
Cox–Snell R-squared: 0.08 Nagelkerke R-squared: 0.11							
Area under the curve: 0.66, CI: 0.573–0.741 (*p* < 0.05)							
Model for men							
BMI			7.701	0.053			
BMI (normal)	−2.970	1.183	6.300	0.012	0.051	0.005	0.522
BMI (overweight)	−2.886	1.054	7.493	0.006	0.056	0.007	0.441
Constant	3.664	1.013	13.086	0.000	39,000		
Model summary: Predicted overall percentage: 79.4%							
Hosmer–Lemeshow test: Chi-square: 0.00 (*p* > 0.05)							
Cox–Snell R-squared: 0.17, Nagelkerke R-squared: 0.26							
Area under the curve: 0.73, CI: 0.623–0.828 (*p* < 0.05)							

Note: SPSS version 27, Wald forward method.

**Table 7:** Binary logistic regression model for non-HDL cholesterol in residents of the city of Trujillo.

General Model	B	Standard Error	Wald	Next.	OR	95% CI for OR	
						Lower	Upper
BMI			15.027	0.002			
BMI (normal)	−1.727	0.455	14.407	0.000	0.178	0.073	0.434
BMI (overweight)	−1.394	0.426	10.691	0.001	0.248	0.108	0.572
Age	0.030	0.013	5.214	0.022	1.030	1.004	1.056
Constant	0.857	0.691	1.537	0.215	2.355		
Model summary: Predicted overall percentage: 74%							
Hosmer–Lemeshow test: Chi-square: 4.954 (*p* > 0.05)							
Cox–Snell R-squared: 0.09, Nagelkerke R-squared: 0.13							
Area under the curve: 0.69, CI: 0.624–0.764 (*p* < 0.05)							
Model for women							
BMI			9.158	0.027			
BMI (normal)	−1.703	0.609	7.817	0.005	0.182	0.055	0.601
BMI (overweight)	−1.017	0.613	2.750	0.097	0.362	0.109	1.203
Age	0.049	0.017	8.000	0.005	1.050	1.015	1.086
Constant	−0.018	0.932	0.000	0.984	0.982		
Model summary: Predicted overall percentage: 76.9%							
Hosmer–Lemeshow test: Chi-square: 2.231 (*p* > 0.05)							
Cox–Snell R-squared: 0.12, Nagelkerke R-squared: 0.17							
Area under the curve: 0.73, CI: 0.64–0.82 (*p* < 0.05)							
Model for men							
BMI			8.564	0.036			
BMI (normal)	−1.861	0.788	5.579	0.018	0.156	0.033	0.729
BMI (overweight)	−1.667	0.598	7.776	0.005	0.189	0.059	0.609
Constant	2.197	0.527	17.380	0.000	9.000		
Model summary: Predicted overall percentage: 73%							
Hosmer–Lemeshow test: Chi-square: 0.00 (*p* > 0.05)							
Cox–Snell R-squared: 0.10, Nagelkerke R-squared: 0.15							
Area under the curve: 0.68, CI: 0.57–0.80 (*p* < 0.05)							

Note: SPSS version 27, Wald forward method.

## Data Availability

The present study was financed by the research support fund of the Vice-Rectorate for Research of the Universidad César Vallejo.

## Supplementary Materials

The following supporting information can be downloaded at: https://www.mdpi.com/article/10.3390/nu16234138/s1, Questionnaires and data collection form S1.
