# Supplementary material for: Predictive Models of Atherogenic Risk in Citizens of Trujillo (Peru) Based on Associated Factors
Source: Nutrients. 2024 Nov 29;16(23):4138. doi: 10.3390/nu16234138 (PMC11644772; doi:10.3390/nu16234138)
Supplement: Supplementary file 1 [file nutrients-16-04138-s001.zip › nutrients-3315992-supplementary.pdf]

EATING HABITS QUESTIONNAIRE

Last Names and First Names: ……………………………………………………………………………………………

Healthy eating habits (Part I)		
1. Eat breakfast 1 = Never<br>2 = Less than 1 per week<br>3 = 1-3 times per week<br>4 = 4-6 times a week 5 = Every day	2. Consume dairy products preferably skimmed (skimmed milk, cottage cheese, fresh cheese or skimmed yogurt)<br>1 = Does not consume<br>2 = Less than 1 time a day<br>3 = 1 serving per day<br>4 = 2 servings per day<br>5 = 3 servings a day	3. Eat fruits (fresh and regular size)<br>1 = Does not consume<br>2 = Less than 1 time per day 3 = 1 serving per day<br>4 = 2 servings per day<br>5 = 3 servings a day
4. Eat vegetables (raw or cooked, portion equivalent to 1 napkin plate) 1 = Do not eat<br>2 = Less than 1 time a day<br>3 = 1/2 serving per day<br>4 = 1 servings per day<br>5 = 2 servings per day	5. Eat fish (fresh/frozen/canned, but not fried)<br>1 = Does not consume<br>2 = Less than 1 time per week 3 = 1 serving per week<br>4 = 2 servings per week<br>5 = 3 servings per week	6. Eat legumes (beans, lentils, peas, chickpeas)<br>1 = Does not consume<br>2 = Less than 1 time per week<br>3 = 1 serving per week<br>4 = 2 servings per week<br>5 = 3 or more servings per week
7. Eat oatmeal or whole grain bread 1 = Do not eat<br>2 = Less than 3 times a week<br>3 = 1 serving per day<br>4 = 2 servings per day<br>5 = 3 servings a day	8. Eat home-cooked food (homemade: stews, carbonadas, casseroles, legumes)<br>1 = Does not consume<br>2 = Less than 1 time per week<br>3 = 1 serving per week<br>4 = 2 servings per week<br>5 = 3 or more servings per week	9. Dinner (lunch + fruit and/or salad) 1 = Never<br>2 = Less than 1 time per week<br>3 = 1-3 times per week<br>4 = 4-6 times a week 5 = Every day

10. Drink water or liquids (herbal water, fruit juices, tea, mate)<br><br>1 = Does not take<br><br>2 = 1 glass a day<br><br>3 = 2 glasses a day<br><br>4 = 3 glasses a day<br><br>5 = 4 or more glasses a day	11. Consumes foods such as meat or poultry 1 = Does not consume<br><br>2 = 1 time every 15 days<br><br>3 = 1 time per week<br><br>4 = 2 times a week<br><br>5 = 3 times a week	12. Eat eggs<br><br>1 = Does not consume<br><br>2 = 1 time every 15 days<br><br>3 = 1 time per week<br><br>4 = 2 times a week<br><br>5 = 3 times a week
13. How many meals do you eat per day? 1 = Less than 1<br><br>2 = 2 meals<br><br>3 = 3 meals<br><br>4 = 4 meals<br><br>5 = 4 meals and snack		
**Unhealthy eating habits (Part II)**		
14. Drink sugary drinks or juices (1 glass of 200 cc serving)<br><br>1 = 3 or more glasses a day 2 = 2 glasses a day<br><br>3 = 1 glass a day<br><br>4 = Less than one glass a day or occasionally<br><br>5 = Does not consume	15. Consume alcoholic beverages (1 glass serving) on the weekend<br><br>1= 3 or more glasses a day 2 = 2 glasses a day<br><br>3 = 1 glass a day<br><br>4 = Less than one glass a day or occasionally<br><br>5 = Does not consume	16. Eat fried foods<br><br>1 = 3 or more servings per week 2 = 2 servings per week<br><br>3 = 1 serving per week<br><br>4 = Occasionally 5 = Does not consume
**Unhealthy eating habits (Part II)**		
17.Usebutter or margarine in the preparation of dough or in frying<br><br>1 = Every day<br><br>2 = 3-5 times per week<br><br>3 = 2-3 times a week<br><br>4 = Less than 1 time per week 5 = Does not use	18. Eat junk food (pizza,sausages or hotdogs,, burgers,empanadas)<br><br>1 = 3 or more servings a day 2 = 2 servings a day<br><br>3 = 1 serving per day<br><br>4 = Less than 1 time per day 5 = Does not consume	19.Eat snacks cookies, cakes, and pastries1 = 3 or more servings per day<br><br>2 = 2 servings per day<br><br>3 = 1 serving per day<br><br>4 = Less than 1 time per day 5 = Does not consume

20. Drink coffee<br><br>1 = 3 or more cups a day 2 = 2 cups a day<br><br>3 = 1 cup a day<br><br>4 = Less than 1 cup a day 5 = Does not consume	21. Add salt to food before tasting it<br><br>1 = Always adds<br><br>2 = Occasionally adds 3 = Does not add	

SOURCE:

Duran, S.; Candia, P.; Pizarro, R. Validación de contenido de la Encuesta de Calidad de Alimentación del Adulto Mayor (ECAAM). Nutr. Hosp. 2017, 34, 1311-1318. https://dx.doi.org/10.20960/nh.1081.

CLASSIFICATION OF HEALTHY EATING HABITS	GOOD HABITS: 52 – 65 POINTS ( )<br><br>BAD HABITS: 13 – 51 POINTS ( )
CLASSIFICATION OF UNHEALTHY EATING HABITS	GOOD HABITS: 31 – 38 POINTS ( )<br><br>BAD HABITS: 8 – 30 POINTS ( )
FOOD QUALITY (SUM OF ITEM I AND II)	GOOD HABITS: 83 - 103 POINTS ( )<br><br>BAD HABITS: 21 - 82 POINTS ( )

INTERNATIONAL PHYSICAL ACTIVITY QUESTIONNAIRE (IPAQ) – MODIFIED

Last Names and First Names:
…….

Dear participant, we are interested in knowing the type of physical activity you do, so we ask you to answer the following questions regarding the time you spent being active in the last 7 days.

-1.- During the last 7 days, what intense physical activities did you mainly do and on how many days?:<br>Lifting weights or heavy objects > 20 kg such as sandbags and/or cement ( )<br>Football, basketball or swimming ( )<br>Bike at a moderate to fast pace, not a walk. ( )<br>Stationary bike with high rhythm ( )<br>Do aerobics ( )<br>digging, hard work with a shovel ( )	
Days per week (please enter number)	
No strenuous physical activity (go to question 3)	☐
2.- How much time in total did you usually spend on intense physical activity on one of those days?	
Please indicate how many hours per day	
Please indicate how many minutes per day	
Don't know/not sure	☐
3- During the last 7 days, what moderate physical activities did you mainly do and on how many days?:<br>Transporting light weights < 20 Kg ( )<br>Ride a bike at a regular speed or at a walking pace ( )<br>Stationary bike in light mode. ( )<br>Tai chi ( )<br>General construction work (painting, plastering, roofing) ( )<br>Gardening ( )<br>Perform modern dance and/or go dancing at the disco ( )	
Days per week (indicate number)	
No moderate physical activity (go to question 5)	☐

4.- How much time in total did you usually spend on moderate physical activity on one of those days?	
Please indicate how many hours per day	
Please indicate how many minutes per day	
Don't know/not sure	☐
5.- During the last 7 days, on how many days did you walk for at least 10 minutes at a time?	
Days per week (please specify number)	
No walking (go to question 7)	☐
6.- How much time in total did you usually spend walking on one of those days?	
Please indicate how many hours per day	
Please indicate how many minutes per day	
Don't know/not sure	☐
7.- During the last 7 days, how much time did you spend sitting during a workday?	
Please indicate how many hours per day	7 h
Please indicate how many minutes per day	
Don't know/not sure	☐

Please note:

1. For walking: 3.3 METs. X 30 min of walking

2. For moderate physical activity: 4 METs. X day per week

3. For vigorous physical activity: 8 METs. X min per day per week

Finally add the totals obtained: walk + AFM + AFV

CLASSIFICATION:

Low	Moderate	High
Does not record physical activity, the physical activity performed is not sufficient to reach the moderate and high categories	3 or more days of vigorous physical activity for at least 20 min per day.<br><br>5 or more days of moderate-intensity physical activity or walking for at least 30 min.<br><br>5 or more days of any combination of light, moderate, or vigorous physical activity that achieve a record of 600 METs-min/week.	3 or more days of vigorous physical activity or accumulating 1,500 METs-min-week.<br><br>7 or more days of any combination of light, moderate, or vigorous physical activity that achieves a record of 3,000 METs-min/week.

Reference:

Carrera, Y. Cuestionario Internacional de actividad física (IPAQ). Revista Enfermería del Trabajo. 2017, 7, 49-54.

Questionnaire on other lifestyles

Last Names and First Names:
…………………………………………………………………

1. How many times a week do you consume alcohol?

a) I don't consume alcohol ( )
b) 1 time per month ( )
c) 1 time per fortnight ( )
d) 1 time a week ( )
e) 2 times a week ( )
f) 3 or more times a week ( )

2. Regarding tobacco consumption

a) I don't smoke ( )
b) I currently smoke ( )
c) I have stopped smoking in the last month ( )

3. If you currently smoke or have quit smoking, how many cigarettes do you smoke?

a) 1 cigarette a week ( )
b) 1 cigarette every other day ( )
c) 1 cigarette per day ( )
d) more than 1 cigarette a day ( )

4. Please indicate how many hours of sleep you get during the day, including naps and going to bed at night.

a) Less than 7h ( )
b) 7 to 9 hours ( )
c) 10 hours or more ( )

Family history

1. Has your father suffered from any of these illnesses:

a) Diabetes mellitus ( )
b) High blood pressure ( )
c) Cardiovascular disease ( )
d) High cholesterol and/or triglycerides ( )
e) None of the diseases mentioned ( )
f) Unknown ( )

2. His mother have suffered from any of these diseases:

a) Diabetes mellitus ( )

b) High blood pressure ( )
c) Cardiovascular disease ( )
d) High cholesterol and/or triglycerides ( )
e) None of the diseases mentioned ( )
f) Unknown ( )

3. Your grandfather has suffered from any of these diseases:

a) Diabetes mellitus ( )

b) High blood pressure ( )
c) Cardiovascular disease ( )
d) High cholesterol and/or triglycerides ( )
e) None of the diseases mentioned ( )
f) Unknown ( )

4. His grandmother have suffered from any of these diseases:

a) Diabetes mellitus ( )

b) High blood pressure ( )
c) Cardiovascular disease ( )
d) High cholesterol and/or triglycerides ( )
e) None of the diseases mentioned ( )
f) Unknown ( )

Physiological and biochemical characteristics

Last Names and First Names: ………………………………………………………….

Age: ………………….

Weight: ………………….Kg

Height: …………………… m

BMI: …………………….Kg/m^2

Abdominal circumference: .... cm

Blood pressure (BP):

Systolic BP: .... mmHg   Diastolic BP: .... mg/dL

Glycemia: .... mg/dL

Lipid Profile

Total Cholesterol: .... mg/dL   Triglycerides: ....mg/dL

HDL: ....mg/dL   LDL: .... mg/dL

Number of risk factors: 0 (.......) 1 (......) 2(......) ≥3 (......)

Atherogenic indicators

COL/HDL (Castelli I risk index): ....

Considering:

Women:

Low Risk < 4.5 ( ) Risk >4.5 ( )

Men:

Low Risk < 5 ( ) Risk >5 ( )

TG/HDL-c : ....

Low CVD Risk: <3 ( ) High Risk: >3 ( )

LDL/HDL (Castelli II risk index): ....

Low risk: <3 ( ) High risk: >3 ( )

COL Non HDL : ....mg/dL

Low risk <130 mg/dL ( ) High risk >130 mg/dL ( )
